# Prevalence and Determinants of Unhealthy Food and Beverage Consumption Among Children Aged 6–23 Months in Mekelle, Northern Ethiopia

**DOI:** 10.1002/fsn3.70729

**Published:** 2025-08-03

**Authors:** Nebyu Daniel Amaha

**Affiliations:** ^1^ Department of Nutrition and Dietetics, School of Public Health, College of Health Sciences Mekelle University Mekelle Tigray Ethiopia

**Keywords:** complementary feeding, Ethiopia, infant and child nutrition, junk food, post‐conflict, sugar‐sweetened beverages, ultra‐processed food, unhealthy foods and beverages

## Abstract

International health authorities, including the World Health Organization, advise against infant consumption of unhealthy foods and beverages (UFB)—products high in sugar, salt, and unhealthy fats but low in micronutrients. Despite global evidence of rising UFB consumption among children, data from post‐conflict, low‐income settings like Mekelle remain limited. This cross‐sectional study assessed UFB consumption among 567 children aged 6–23 months in three randomly selected health facilities in Mekelle, northern Ethiopia, following the 2020–2022 war. Caregiver‐reported 24‐h recall revealed that 71.8% of children consumed at least one UFB, with sugar‐sweetened beverages being most common (62.9%), followed by sweets (41.8%), soft drinks (18.2%), and fried foods (9.2%). UFB consumption increased with age, peaking at 81.1% among 18‐ to 23‐month‐olds. Compared to children aged 6–11 months, those aged 12–17 months (Adjusted Odds Ratio (AOR): 2.3, 95% CI: 1.46–3.64, *p* < 0.001) and 18–23 months (AOR = 4.14, 95% CI: 2.36–7.26, *p* < 0.001) had significantly higher odds of UFB consumption. Children from high‐income households were 51% less likely to consume UFBs than those from low‐income households (AOR 0.49, 95% CI: 0.28–0.87, *p =* 0.014). Birth order influenced intake, with third/later‐born children nearly four times more likely to consume UFBs than first‐borns (AOR: 3.78, 95% CI: 1.19–11.98, *p =* 0.024). Larger household sizes showed protective effects, with four‐member (AOR: 0.31, 95% CI: 0.10–0.96, *p =* 0.04) and five‐ to eight‐member households (AOR: 0.28, 95% CI: 0.09–0.87, *p =* 0.028) having lower consumption than smaller households. The high UFB consumption in postwar Mekelle poses public health risks, particularly among older children and those from low‐income households. Targeted nutrition education, stricter UFB regulation, and improved access to healthy foods are essential to address this issue and promote healthier feeding practices.

## Graphical abstract

This cross‐sectional study examined unhealthy food and beverage (UFB) consumption among 567 children aged 6–23 months in postwar Mekelle, Ethiopia. Findings revealed that 71.8% of children consumed at least one UFB, with sugar‐sweetened beverages being the most common (62.9%), followed by sweets (41.8%), soft drinks (18.2%), and fried foods (9.2%). Older child age, low household income, and higher birth order were significantly associated with increased UFB intake.

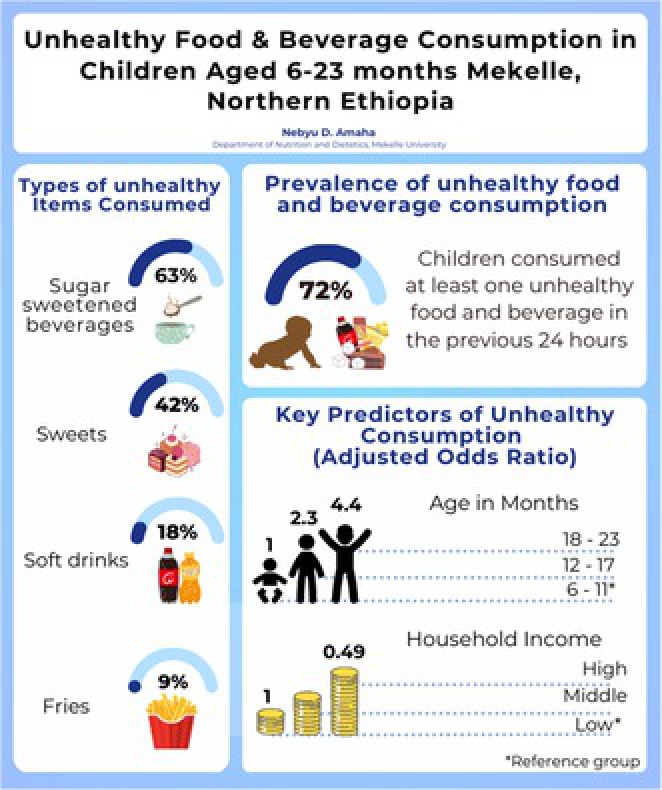

## 1 Introduction

Unhealthy foods and beverages (UFBs) are foods high in added sugars, salt, and unhealthy fats. These include sugar‐sweetened beverages, non‐sugar sweeteners, and processed snacks that are energy‐dense but nutrient‐poor. Regular consumption of UFBs is linked to undernutrition, overweight, and adverse cardiovascular and metabolic outcomes (Lutter et al. 2021; WHO 2023b). Although the World Health Organization recommends against the consumption of UFBs by infants and young children, these food products are widely consumed due to their high palatability, affordability compared to nutritious foods, convenience, and aggressive marketing (Green et al. 2019; Van der Kooy et al. 2023).

Economic development in many low‐ and middle‐income countries (LMICs) has driven significant dietary changes. Increased availability and affordability of processed foods have enabled families to purchase these products more frequently (Mekonen et al. 2024; Mwesigwa and Naidoo 2024). This shift has displaced traditional foods like whole grains and legumes with Westernized diets high in added sugars, unhealthy fats, and refined carbohydrates (Van der Kooy et al. 2023). Urbanization has further accelerated this transition by providing greater access to processed foods and promoting convenience‐driven lifestyles at the expense of nutritional quality (Huffman et al. 2014).

The prevalence of UFB consumption among children is rising globally, particularly in LMICs. A study across five sub‐Saharan African countries reported that 62.4% of children aged 6–23 months exhibited unhealthy feeding practices (Tekeba et al. 2025). For example, in Bandung City, Indonesia, 81.6% of children aged 6–35 months consumed commercially produced snack foods and sugar‐sweetened beverages (Green et al. 2019). Similarly, in China, the proportion of children consuming sweet beverages increased significantly from 2013 to 2016/2017, reaching 51.7% (Wang et al. 2023).

In Ethiopia, high UFB consumption rates have been reported in various regions. In Gondar, Northwest Ethiopia, 64% of children consumed UFB, while in Southwest Ethiopia, the rate was 44.3% (Debessa et al. 2023; Jemere et al. 2023). However, little is known about UFB consumption in postwar urban settings such as Mekelle, where the 2‐year war (2020–2022) and its social and economic changes may influence dietary behaviors. This study aimed to determine the prevalence of UFB consumption and identify associated factors among infants and young children in Mekelle, northern Ethiopia.

## 2 Methods

### 2.1 Study Setting

This study was conducted in Mekelle Special Zone, encompassing Mekelle City, the regional capital of the Tigray region. Tigray, one of Ethiopia's 12 regional states, experienced significant upheaval during the 2‐year war (2020–2022) between the Tigray Regional Government and the Ethiopian Federal Government. This study was carried out 2 years after the signing of the Pretoria Agreement on November 2, 2022. Located 780 km north of Addis Ababa, the capital of Ethiopia, Mekelle is subdivided into seven subcities and has an estimated population of approximately 612,000 as of 2024 (Macrotrends 2024). The city's healthcare infrastructure includes 5 hospitals and 11 health centers (Gebregziabher et al. 2022). The timing of data collection (2024) reflects a critical post‐war recovery period.

### 2.2 Study Design and Population

This study employed a facility‐based cross‐sectional design and was conducted between November and December 2024 in Mekelle. The study population comprised mothers/caregivers with children aged 6–23 months who attended the Extended Program on Immunization (EPI) services at one of the randomly selected health centers. Data on sociodemographic characteristics and dietary intake were collected via face‐to‐face interviews using a pretested and standardized questionnaire (Kennedy et al. 2011).

### 2.3 Sample Size and Sampling Procedure

The sample size was determined using a double population proportion formula by assuming alpha = 0.05, power = 80%, and taking maternal education, household income, and child age as exposure factors from the 2019 Ethiopian Mini Demographic and Health Survey (EDHS 2021). The final calculated sample size was 584 (Table S1). Then, a multistage sampling technique was used to identify the participants of this study. In the first sampling stage, three subcities were randomly selected from the six subcities in Mekelle city. Then, during the second stage, one health center was randomly selected from the available health centers within the subcity. The final sample size from each health center was used based on the number of children visiting the health centers and allocated proportionally. Children who came with non‐consenting caregivers and children with severe chronic illnesses or congenital anomalies were excluded from the study. In cases where children were accompanied by someone other than their primary caregiver, only respondents who could confirm they were regularly involved in the child's feeding were interviewed.

### 2.4 Study Variables

The outcome variable is the consumption of unhealthy foods and beverages, defined as the intake of at least one of the following in the previous 24 h: fries, sweets, soft drinks, or sugar‐sweetened beverages (WHO 2023a).

- “Sweets” refers to sugar confections (caramel, chocolate, candies, and toffee), sweet baked/fried confections (cakes, cookies, sweet biscuits, sweet pies, and sweet pastries), or frozen desserts/treats (ice cream and gelato).
- “Fries” refers to French fries or crisps/potato chips.
- “Sugar‐sweetened beverages (SSB)” included tea, coffee, or herbal drinks with added sugar.
- “Soft drinks” include Coca‐Cola, Fanta, Sprite, Mirinda, Pepsi, and other carbonated energy drinks.

Predictor variables included the child's age, child sex, birth order of the child, parental educational level and type of occupation, maternal age, marital status, maternal internet use, family size, and household income in Ethiopian birr. The continuous variables of maternal age, child age, birth order, and family size were categorized into meaningful groups with similar percentages for each group. Household monthly income was divided into low, middle, and high‐income terciles (equal 33.3% divisions of the sample).

### 2.5 Data Collection

Data were collected using a structured, validated questionnaire adapted from FAO and EDHS tools (EDHS 2016; Kennedy et al. 2011). The questionnaire was initially developed in English, translated into Tigrigna (the local language), and then back‐translated to ensure consistency and accuracy. Face‐to‐face interviews were conducted with mothers/caregivers, focusing on dietary intake over the previous 24 h and relevant sociodemographic factors. Community health workers were recruited to conduct the data collection. Daily supervision was implemented to ensure the accuracy and completeness of the data.

### 2.6 Data Analysis

Data were entered into Microsoft Excel, cleaned, and then analyzed using STATA statistical software version 13. Missing data were assessed separately for food consumption and sociodemographic variables. Little's MCAR test confirmed missing food data (soft drinks [*n* = 3], beverages [*n* = 7], sweets [*n* = 3], fries [*n* = 9]) were completely at random (*χ* ^2^ = 6.79, *p* = 0.659), justifying listwise deletion (final *n* = 567). For sociodemographic variables (maternal age [*n* = 11], paternal education/employment [*n* = 30], income [*n* = 18], internet use [*n* = 6]), the significant MCAR test (*χ* ^2^ = 623.15, *p* < 0.001) indicated non‐random missingness, addressed via median/mode imputation. Sensitivity analyses (complete‐case vs. imputed) confirmed robustness (Table S3).

Descriptive statistics were used to summarize the sociodemographic characteristics of the respondents and consumption of UFB. Categorical variables were presented as frequencies and percentages, while continuous variables were presented as means and standard deviations. Chi‐square was used to assess associations between categorical variables. To address potential confounding, we included key sociodemographic variables in the multivariable logistic regression model. To further investigate the relationships between predictors and UFB consumption, we performed subgroup analyses stratified by child age based on previous evidence from two studies in Ethiopia (Debessa et al. 2023; Jemere et al. 2023), suggesting that unhealthy food consumption patterns differ significantly across different age groups. Age was divided into three categories: 6–11, 12–17, and 18–23 months. Each subgroup was analyzed separately to explore potential effect modifications by age. Sensitivity analysis was carried out by using the complete case analysis, which excluded all participants with missing data.

## 3 Results

The sociodemographic profile showed that children had a mean age of 13.6 months, and mothers averaged 28.6 years. Households typically had four members, with a median child order of 2, and an average monthly income of 10,561 Ethiopian Birr (ETB). Of the children, 54.3% were female, and 91.8% of mothers were married. Regarding education, 29.97% of mothers had less than 8 years of schooling, 35.8% completed high school, and 34.25% had over 12 years of schooling, while 54.3% of fathers had more than 12 years of education. Most mothers were homemakers (57.4%), fathers were primarily in government jobs (39.7%), and 59.8% of mothers reported internet use (Table 1).

**TABLE 1:** Sociodemographic characteristics of the respondents and their children aged 6–23 months in Mekelle (*n* = 584).

Continuous variables	Mean	SD	Min–Max
Age of child (months)	13.6	4.8	6–23
Age of mother (years)	28.6	5.12	17–42
Household numbers	4^a^	1.2	1–8
Child order	2^a^	1.02	1–6
Household income in ETB	10,561	10,207	0–100,000
Categorical variables	Categories	Frequency	%
Child sex	Male (%)	267	45.7
	Female	317	54.3
Marital status	Married	536	91.8
	Others	48	8.25
Maternal education years	< 8 years	175	29.97
	9–12 years	209	35.79
	> 12 years	200	34.25
Paternal education years	< 8 years	144	24.7
	9–12 years	123	21.1
	> 12 years	317	54.3
Maternal job	Unemployed	335	57.36
	Government	141	24.14
	Other	108	18.49
Paternal job	Government	232	39.7
	Merchant	149	25.5
	Others	203	34.8
Maternal internet use	Yes	349	59.8
	No	235	40.2

Abbreviation: SD, standard deviation.

^a^ Median.

### 3.1 Unhealthy Food and Beverage Consumption

Of the 567 children aged 6–23 months, 71.8% consumed at least one type of UFB in the previous day. Sugar‐sweetened beverages (62.9%) were the most common, followed by sweets (41.8%), soft drinks (18.2%), and fried foods (9.2%). The chi‐square test showed that UFB consumption was significantly associated with child age (*p* < 0.001), with the highest prevalence among children aged 12–17 months (37.3%). Maternal education (*p* = 0.006), paternal education (*p* < 0.001), and household income (*p* < 0.001) also showed an association with UFB consumption. Higher rates of overall consumption were observed among children of mothers with 8–12 years of education (37.6%), fathers with over 12 years of education (47.2%), and low‐income households (37.6%). Maternal internet access (*p* = 0.003) was another significant factor, as children of mothers with internet access had higher UFB consumption (55.3%) than those without (44.7%) (Table 2).

**TABLE 2:** Number of children aged 6–23 who consumed sugar‐sweetened beverages and unhealthy foods on the previous day in Mekelle (*n* = 567).

			Soft drink	Sweets	Fries	SSB^a^	Overall (UFB^b^)	*p* ^c^
			*n* (%)	*n* (%)	*n* (%)	*n* (%)	*n* (%)	
Child's age in months	6–11	228	31 (30.1)	70 (29.5)	7 (13.5)	122 (34.2)	139 (34.2)	< 0.001
	12–17	196	44 (42.7)	101 (42.6)	21 (40.4)	134 (37.5)	152 (37.3)	
	18–23	143	28 (27.2)	66 (27.8)	24 (46.2)	101 (28.3)	116 (28.5)	
Child sex	Male	260	42 (40.8)	103 (43.5)	21 (40.4)	157 (44.0)	181 (44.5)	0.337
	Female	307	61 (59.2)	134 (56.5)	31 (59.6)	200 (56.0)	226 (55.5)	
Child order	First	192	39 (37.9)	74 (31.2)	16 (30.8)	119 (33.3)	139 (34.2)	0.452
	Second	209	32 (31.1)	81 (34.2)	19 (36.5)	128 (35.9)	144 (35.4)	
	Third and above	166	32 (31.1)	82 (34.6)	17 (32.7)	110 (30.8)	124 (30.5)	
Maternal age	17–25	165	35 (34.0)	68 (28.7)	16 (30.8)	103 (28.9)	126 (31.0)	0.197
	26–35	346	59 (57.3)	141 (59.5)	30 (57.7)	216 (60.5)	239 (58.7)	
	> 36	56	9 (8.7)	28 (11.8)	6 (11.5)	38 (10.6)	42 (10.3)	
Household members	1–3	216	39 (37.9)	88 (37.1)	17 (32.7)	141 (39.5)	163 (40.0)	0.226
	4	199	31 (30.1)	77 (32.5)	20 (38.5)	120 (33.6)	135 (33.2)	
	5–8	152	33 (32.0)	72 (30.4)	15 (28.8)	96 (26.9)	109 (26.8)	
Marital status	Married	522	100 (97.1)	222 (93.7)	48 (92.3)	334 (93.6)	378 (92.9)	0.333
	Others	45	3 (2.9)	15 (6.3)	4 (7.7)	23 (6.4)	29 (7.1)	
Maternal educational level	< 8	170	28 (27.2)	81 (34.2)	19 (36.5)	115 (32.2)	131 (32.2)	0.006
	8–12	203	37 (35.9)	93 (39.2)	17 (32.7)	134 (37.5)	153 (37.6)	
	> 12	194	38 (36.9)	63 (26.6)	16 (30.8)	108 (30.3)	123 (30.2)	
Maternal occupation	Homemaker	325	56 (54.4)	135 (57.0)	27 (51.9)	209 (58.5)	239 (58.7)	0.082
	Government	138	24 (23.3)	49 (20.7)	15 (28.8)	82 (23.0)	89 (21.9)	
	Other	104	23 (22.3)	53 (22.4)	10 (19.2)	66 (18.5)	79 (19.4)	
Paternal educational level	< 8	146	18 (17.5)	76 (32.1)	18 (34.6)	98 (27.5)	113 (27.8)	< 0.001
	8–12	123	25 (24.3)	56 (23.6)	12 (23.1)	92 (25.8)	102 (25.1)	
	> 12	298	60 (58.3)	105 (44.3)	22 (42.3)	167 (46.8)	192 (47.2)	
Paternal occupation	Merchant	210	35 (40.7)	76 (40.2)	16 (39.0)	100 (35.2)	115 (35.9)	0.134
	Government	152	38 (44.2)	79 (41.8)	15 (36.6)	127 (44.7)	139 (43.4)	
	Others	205	13 (15.1)	34 (18.0)	10 (24.4)	57 (20.1)	66 (20.6)	
Household income level	Low	195	26 (25.2)	90 (38.0)	17 (32.7)	135 (37.8)	153 (37.6)	< 0.001
	Medium	206	42 (40.8)	79 (33.3)	19 (36.5)	117 (32.8)	137 (33.7)	
	High	166	35 (34.0)	68 (28.7)	16 (30.8)	105 (29.4)	117 (28.7)	
Maternal internet use	Yes	336	66 (64.1)	133 (56.1)	31 (59.6)	192 (53.8)	225 (55.3)	0.003
	No	231	37 (35.9)	104 (43.9)	21 (40.4)	165 (46.2)	182 (44.7)	
	Total	*N* = 567	103 (18.2)	237 (41.8)	52 (9.2)	357 (62.9)	407 (71.8)	

^a^ SSB: Sugar‐sweetened beverages.

^b^ UFB: unhealthy foods and beverages.

^c^ *p*‐value of chi‐square test.

**FIGURE 1:**
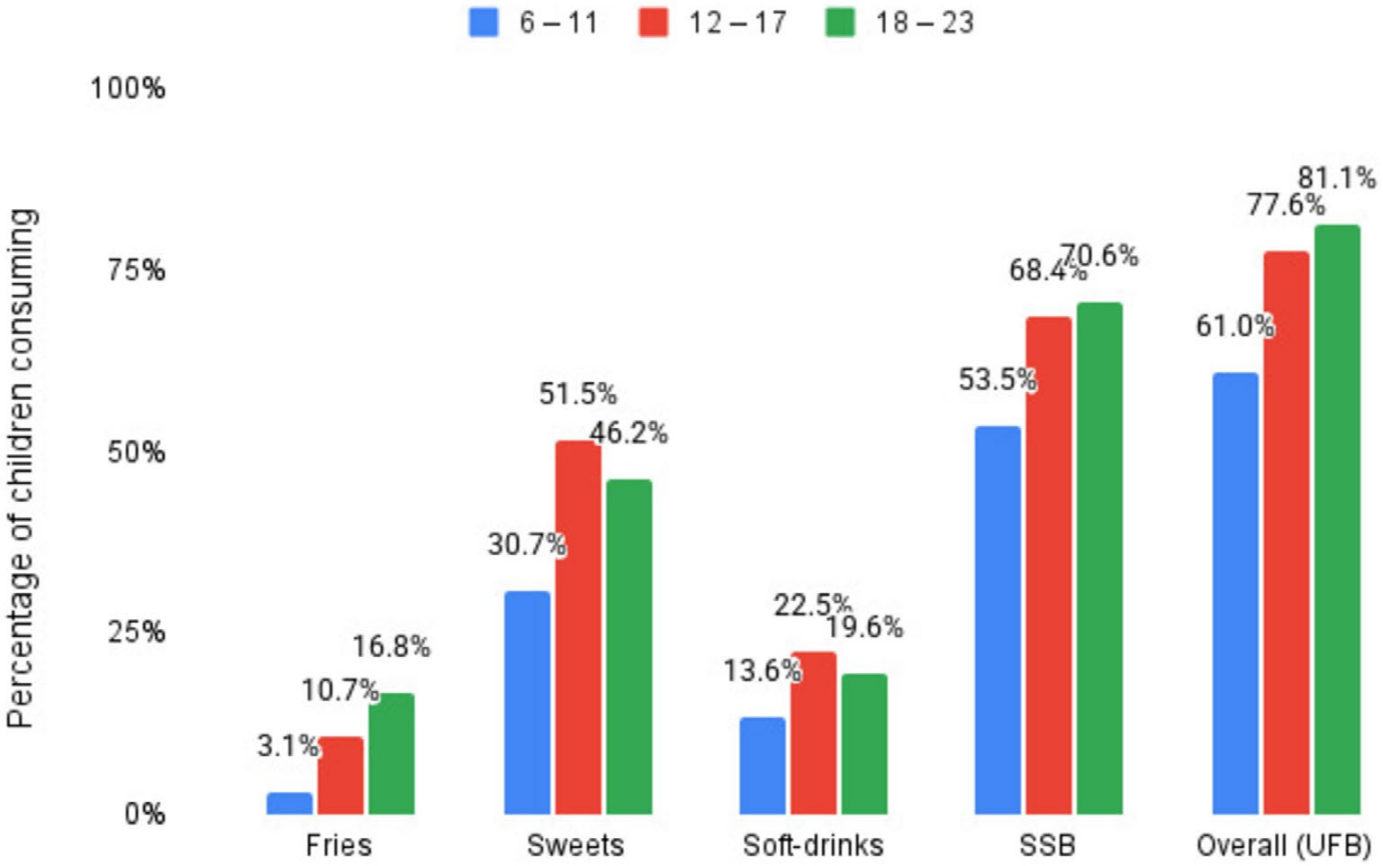
Age‐disaggregated consumption patterns of unhealthy foods and beverages (UFB) and sugar‐sweetened beverages (SSB) among children aged 6–23 months in Mekelle, northern Ethiopia.

The overall consumption of UFB increased with age, highest in the 18–23‐month group (81.1%), followed by the 12‐ to 17‐month group (77.6%) and the 6‐ to 11‐month group (61.0%). Fries consumption rose from 3.1% in the youngest group to 10.7% and 16.8% in the older groups. Sweets consumption peaked in the 12‐ to 17‐month group at 51.5%, compared to 30.7% in the youngest and 46.2% in the oldest groups. Soft drink consumption was 13.6%, 22.5%, and 19.6% across the three age groups, respectively. Sugar‐sweetened beverages showed a significant increase from 53.5% in the youngest group to 68.4% and 70.6% in the older groups (Figure 1).

### 3.2 Factors Associated With Sweet Beverage Consumption

Child age was significantly associated with UFB consumption, with children aged 12–17 and 18–23 months being more likely to consume UFBs compared to those aged 6–11 months (AOR: 2.3, 95% CI: 1.46–3.64, *p* < 0.001; AOR: 4.14, 95% CI: 2.36–7.26, *p* < 0.001, respectively). Higher birth order also increased the likelihood of UFB consumption, with children of third or higher birth order having nearly four times the odds compared to first‐borns (AOR: 3.78, 95% CI: 1.19–11.98, *p* = 0.024). Household composition showed that children from households with 4 members (AOR: 0.31, 95% CI: 0.10–0.96, *p* = 0.042) or 5–8 members (AOR: 0.28, 95% CI: 0.09–0.87, *p* = 0.028) were less likely to consume UFBs than those from smaller households with 1–3 members. Additionally, children of unmarried mothers had significantly lower odds of UFB consumption compared to those of married mothers (AOR: 0.26, 95% CI: 0.11–0.62, *p* = 0.002). Finally, higher household income was associated with reduced odds of UFB consumption, with children from high‐income households being less likely to consume UFBs than those from low‐income households (AOR: 0.49, 95% CI: 0.28–0.87, *p* = 0.014) (Table 3).

**TABLE 3:** Binary and multivariable logistic regression analysis of the factors associated with unhealthy foods and beverages consumption in the previous 24 h among children aged 6–23 months in Mekelle, northern Ethiopia (*n* = 567).

		Unadjusted		Adjusted	
		COR^a^	*p*	AOR^b^	*p* ^c^
Child age in months	6–11	Ref		Ref	Ref
	**12–17** ^d^	2.21 (1.44–3.39)	< 0.001	**2.3 (1.46–3.64)**	**< 0.001**
	**18–23** ^d^	2.75 (1.67–4.52)	< 0.001	**4.14 (2.36–7.26)**	**< 0.001**
Child sex	Male	Ref		Ref	
	Female	1.22 (0.84–1.76)	0.292	1.14 (0.76–1.7)	0.531
Childbirth order	First	Ref		Ref	
	Second	0.84 (0.55–1.3)	0.443	2.5 (0.79–7.92)	0.12
	**Third and above** ^d^	1.13 (0.7–1.8)	0.623	**3.78 (1.19–11.98)**	**0.024**
Maternal age	17–25	Ref		Ref	
	26–35	0.69 (0.45–1.06)	0.089	0.69 (0.38–1.24)	0.213
	> 36	0.93 (0.46–1.88)	0.836	0.67 (0.26–1.76)	0.417
Household members	1–3	Ref		Ref	
	**4** ^d^	0.69 (0.45–1.05)	0.085	**0.31 (0.1–0.96)**	**0.042**
	**5–8** ^d^	0.82 (0.52–1.32)	0.42	**0.28 (0.09–0.87)**	**0.028**
Marital status	Married	Ref		Ref	
	**Other** ^d^	0.69 (0.36–1.31)	0.257	**0.26 (0.11–0.62)**	**0.002**
Maternal education in years	< 8	Ref		Ref	
	8–12	0.91 (0.56–1.47)	0.703	1.39 (0.76–2.54)	0.288
	> 12	0.52 (0.33–0.82)	0.005	1.12 (0.53–2.35)	0.768
Maternal occupation	Homemaker	Ref		Ref	
	Gov't	0.65 (0.43–1)	0.051	1.03 (0.55–1.9)	0.935
	Other	1.14 (0.68–1.9)	0.623	1.39 (0.76–2.53)	0.285
Paternal education in years	< 8	Ref		Ref	
	8–12	1.42 (0.77–2.61)	0.261	1.29 (0.64–2.59)	0.471
	> 12	0.53 (0.34–0.83)	0.006	0.7 (0.33–1.48)	0.355
Paternal occupation	Government	Ref		Ref	
	Merchant	1.59 (0.99–2.53)	0.053	1.34 (0.74–2.43)	0.339
	Other	1.5 (0.98–2.3)	0.06	1.1 (0.61–2)	0.753
Household income tercile	Low	Ref		Ref	
	Medium	0.55 (0.35–0.87)	0.011	0.6 (0.36–1)	0.052
	**High** ^d^	0.44 (0.27–0.71)	0.001	**0.49 (0.28–0.87)**	**0.014**
Maternal internet use	Yes	Ref		Ref	
	No	1.83 (1.24–2.7)	0.002	1.49 (0.87–2.55)	0.142

^a^ COR, Crude Odds Ratio.

^b^ AOR, Adjusted Odds Ratio.

^c^ *p*‐value.

^d^ Bold values are statistically significant.

Although maternal education, occupation, and internet use were not statistically significant in the adjusted model, subgroup analysis revealed age‐specific associations. For children aged 12–17 months, both marital status and maternal internet use were significant predictors of UFB consumption, whereas for children aged 6–11 months, family size and household income were significant. No predictors were significant for the 18‐ to 23‐month subgroup (Table S2).

The AORs for most predictors were consistent between the imputed and complete case analyses, though some differences in magnitude and significance emerged. For child birth order, the “second child” category, initially non‐significant in the imputed data, became significant in the complete case analysis (AOR = 4.67, *p* = 0.048), and the “third & above” category showed a stronger association (AOR = 4.15, *p* = 0.037 vs. AOR = 3.78, *p* = 0.024). The inverse relationship for households with four members was more pronounced in the complete case analysis (AOR = 0.15, *p =* 0.013 vs. AOR = 0.31, *p* = 0.042). While high household income remained significant in both analyses, its association was slightly weaker in the complete case analysis (AOR = 0.55, *p* = 0.046 vs. AOR = 0.49, *p* = 0.014). Marital status, significant in the imputed analysis (AOR = 0.26, *p =* 0.002), lost significance in the complete case analysis (AOR = 0.44, *p* = 0.286). However, child age consistently demonstrated strong associations across both analyses, confirming its robustness as a key determinant (Table S3).

## 4 Discussion

This study aimed to explore the prevalence of UFB consumption and the factors associated with UFB consumption among children aged 6–23 months in postwar Mekelle, northern Ethiopia. The findings revealed a high prevalence (71.8%) of UFB consumption, with notable variations by child age and specific sociodemographic factors. Soft‐drink consumption was reported by 18.2% of children, sweetened beverages by 62.9%, sweets by 41.8%, and fries by 9.2%. Higher child age and birth order, lower household income, and smaller family size were significantly associated with increased UFB consumption.

Our study revealed a high prevalence of 71.8% of unhealthy foods and beverages (UFB); however, it aligns with a broader pattern of high UFB consumption in certain LMICs. It is comparable with the 62.4% from five sub‐Saharan countries (Tekeba et al. 2025), 63.7% in Gondar City, Northwest Ethiopia (Jemere et al. 2023), and 74.1% from Nepal (Pries et al. 2016). These results show that the increasing UFB consumption in LMICs due to their cheaper cost and higher availability poses a challenge in providing healthy complementary foods. Alarmingly, up to 42.6% of daily energy intake at preschool age in low‐income settings in Brazil has been attributed to UFB consumption (Rauber et al. 2015). This widespread consumption of unhealthy foods is a significant public health concern, as it may contribute to the displacement of nutritious foods and the development of a double burden of malnutrition (Rousham et al. 2022; Tizazu et al. 2024; Van der Kooy et al. 2023). Addressing this issue requires targeted interventions that consider key factors such as socioeconomic status, nutrition education, and the specific types of unhealthy foods consumed. Policy changes are also critical, including regulating the marketing of unhealthy foods, implementing warning labels, and subsidizing nutritious food options to encourage healthier dietary practices (Mwesigwa and Naidoo 2024; Nordhagen et al. 2019).

### 4.1 Child Factors

Older children were significantly more likely to consume UFB, with those aged 18–23 months being four times more likely (AOR = 4.42, 95% CI: 2.36–7.26, *p* < 0.001) than those aged 6–11 months. Similar age‐related trends were observed in sub‐Saharan Africa (Mekonen et al. 2024), Cambodia and Nepal (Pries et al. 2019). As children age, their dietary exposure broadens, often including less healthy options (Saldan and Mello 2019). Early exposure to unhealthy foods may create lasting taste preferences (Pries et al. 2019). Mekonen et al. suggested that older children's integration into family meals, which often include unhealthy food items, contributes to this trend (Mekonen et al. 2024), while Jemere et al. suggested that evolving feeding practices, shaped by convenience and cultural norms, might drive higher UFB consumption among older children (Jemere et al. 2023). Contrary to our findings, Jemere et al. in Gondar, Ethiopia, reported that older children were less likely to consume UFB (AOR = 0.53, 95% CI: 0.34–0.74, *p* < 0.05) (Jemere et al. 2023). This discrepancy may be due to differences in parental feeding practices, food availability, or cultural attitudes toward child feeding across regions.

Third and above‐born children were 3.8 times more likely (AOR = 3.78, 95% CI: 1.19–11.98, *p* = 0.024) to consume UFB when compared with the first‐born child. Higher birth order children are significantly more likely to consume fewer food groups (OR 1.11) and less often eat fruits and vegetables (OR 1.09) (Howell et al. 2016). Families with multiple children may experience time and resource constraints, potentially leading to increased reliance on convenient, but less healthy, options like UFBs to save time and effort in meal preparation.

### 4.2 Maternal Factors

While child‐level factors showed strong associations, maternal characteristics revealed more complex patterns. Higher parental education was initially associated with lower odds of UFB consumption among children in Mekelle. Mothers with more than 12 years of education were 48% less likely (Crude Odds Ratio (COR) = 0.52, 95% CI: 0.33–0.82, *p* = 0.005) and fathers 47% less likely (COR = 0.53, 95% CI: 0.25–0.70, *p* = 0.001) to feed their children UFB compared to those with less than 8 years of schooling. However, this association lost significance after adjusting for confounders, indicating potential mediation by other factors. Similar findings have been reported by (Mekonen et al. 2024) that mothers with secondary or higher education were significantly less likely to feed their children UFB. However, Green et al. (2019) and Saldan and Mello (2019) found that lower maternal education was linked to increased consumption of snack foods and sweets. In contrast, some studies reported that higher maternal education was sometimes associated with increased UFB consumption (Mwesigwa and Naidoo 2024; Nordhagen et al. 2019), reflecting the complexity of this relationship, which may vary based on context, food types, and socioeconomic factors.

Contrary to trends observed in other African contexts (Tekeba et al. 2025), unmarried mothers in our study were 74% less likely to provide UFB to their children (AOR = 0.26, 95% CI: 0.11–0.62, *p =* 0.002) when compared with married ones. Unmarried mothers may feel a greater sense of individual responsibility for their children's nutrition and health. Without a partner, they might prioritize healthier feeding practices to ensure their child's well‐being. Conversely, married mothers showed higher odds of providing UFBs (AOR = 3.85), potentially due to shared childcare responsibilities that encourage more relaxed feeding practices. Spousal involvement in food purchasing decisions may favor convenient, processed options, particularly in dual‐income households where time constraints are greater.

### 4.3 Household Factors

Beyond individual caregiver traits, household structural factors further shaped UFB consumption. Children from households with four or five to eight members were 69% and 72% less likely to consume UFB, respectively, compared to those from smaller households with one to three members. While (Green et al. 2019) found no significant link between household size and SSB consumption, Jemere et al. reported that children in larger families were more likely to consume unhealthy foods (Jemere et al. 2023). Limited resources in larger families may reduce the purchase of costly, processed foods, leading to a reliance on affordable staples and home‐cooked meals. Intra‐household food distribution might also prioritize healthier options. However, as (Jemere et al. 2023) noted, larger families might foster a desire for unhealthy foods, reflecting the nuanced relationship between household size and dietary habits.

The relationship between household income and UFB consumption shows varying trends. In our study, children from the highest wealth tercile were 51% less likely to consume UFB compared to those in the lowest income tercile (AOR = 0.49, 95% CI: 0.28–0.87, *p* = 0.014), aligning with findings from (Nordhagen et al. 2019; Pries et al. 2019), who reported similar negative associations. Pries and Chiwila et al. also reported higher UFB consumption among children from lower socioeconomic households, with children in the lowest wealth tercile being 1.5 times more likely to consume commercial snack foods (Chiwila et al. 2024; Pries et al. 2017; Pries et al. 2019). However, Mekonen et al. observed that children from wealthier households were 1.20 times more likely to consume UFB (Mekonen et al. 2024). These contrasting findings may reflect the complexity of socioeconomic factors influencing UFB consumption. Lower socioeconomic status often limits access to diverse, nutritious foods, making cheaper, processed options more appealing. Pries et al. noted that children in the poorest households derived a larger share of energy from non‐breastmilk foods like biscuits and snacks (Pries et al. 2019), which highlights economic constraints as a key factor. Additionally, socioeconomic status is commonly measured through proxies like dwelling characteristics and access to basic services, which may influence dietary behaviors differently across settings (Chiwila et al. 2024; Salem et al. 2022), predisposing low‐income families to have higher access to less nutritious and cheaper foods.

The subgroup analysis revealed that family size and household income significantly influenced UFB consumption only in the youngest age group (6–11 months), highlighting the role of household dynamics and resources during early infancy. Larger families may resort to convenience foods due to time and resource constraints, while lower‐income households might opt for cheaper, less nutritious options. In the 12–17 months group, marital status became significant, suggesting that changes in family structure, such as single or separated caregivers, could affect feeding practices as children develop independent eating habits. By 18–23 months, none of these predictors remained significant, indicating that factors like cultural norms, caregiver behaviors, and children's growing autonomy and preferences may play a more substantial role in influencing UFB consumption at this stage.

This study has several limitations that should be considered when interpreting the findings. First, the cross‐sectional nature of the study precludes establishing causality between the identified factors and UFB consumption. Second, the dietary data were based on a single 24‐hr recall, which may not reflect usual intake and is prone to recall bias. This method also does not account for day‐to‐day or seasonal variations in dietary intake, potentially misrepresenting children's habitual consumption patterns. Third, despite efforts to minimize recall and social desirability bias during interviews, mothers may have under‐ or over‐reported UFB consumption. Fourth, as noted by the reviewers, employing community health workers as data collectors, while beneficial for community engagement and local knowledge, may have introduced observer bias or social desirability bias due to their dual role as service providers. Although they received training and were supervised throughout the data collection process, this potential bias should be acknowledged. Fifth, the study took place in a post‐conflict urban setting, which may not reflect rural realities or fully capture the nutritional impact of the war across different population groups. Access to health services, food markets, and household dynamics may differ significantly in rural areas. We recommend future research using a Food Frequency Questionnaire (FFQ) alongside the 24‐h recall to provide a more comprehensive picture of dietary habits over time. Moreover, future studies should also employ larger samples to adequately power subgroup analyses, and further research is warranted to explore how these determinants operate across a wider range of cultural and socioeconomic contexts.

## 5 Conclusion

This study highlights a high prevalence of UFB consumption among young children in postwar Mekelle, with 71.8% of children aged 6–23 months consuming at least one UFB. The most commonly consumed items were sugar‐sweetened beverages (62.9%) and sweets (41.8%). UFB consumption increased significantly with age, peaking at 81.1% among 18‐ to 23‐month‐olds, suggesting that dietary habits worsen as children grow older. Socioeconomic factors played an important but complex role—children from higher‐income households were significantly less likely to consume UFBs (AOR = 0.49), while those in larger families (> 4 members) showed reduced consumption compared to smaller households. Notably, unmarried mothers were substantially less likely to provide UFBs than married mothers (AOR = 0.26), indicating that household structure influences feeding practices. These findings emphasize the need for targeted interventions that address age‐specific dietary transitions, improve access to nutritious foods for low‐income families, and consider family dynamics in nutrition education programs, particularly in post‐conflict settings where food systems remain vulnerable. Integrating UFB education into routine EPI visits could leverage existing healthcare touchpoints to promote healthier feeding practices during critical developmental windows.

## Author Contributions

**Nebyu Daniel Amaha:** conceptualization (lead), data curation (lead), formal analysis (lead), investigation (lead), methodology (lead), software (lead), validation (lead), visualization (lead), writing – original draft (lead), writing – review and editing (lead).

## Ethics Statement

Ethical approval was obtained from the Institutional Review Board (IRB) of the College of Health Sciences, Mekelle University (Approval No: MU‐IRB 2376/2024). Verbal informed consent was obtained from all participants before data collection. Participants were assured of the confidentiality of their responses and the voluntary nature of their participation.

## Conflicts of Interest

The author declares no conflicts of interest.

## Supporting information


Data S1.

## Data Availability

The data that support the findings of this study are available on request from the corresponding author. The data are not publicly available due to privacy and ethical restrictions.
