# Supplementary material for: Prevalence and Determinants of Unhealthy Food and Beverage Consumption Among Children Aged 6–23 Months in Mekelle, Northern Ethiopia
Source: Food Sci Nutr. 2025 Aug 3;13(8):e70729. doi: 10.1002/fsn3.70729 (PMC12318821; doi:10.1002/fsn3.70729)
Supplement: Supplementary file 1 — Data S1. [file FSN3-13-e70729-s001.docx]

**Table S1 Sample size determination using the double population proportion formula**

| Factors | % of exposed | Power | % of unexposed | OR | Sample Size | 10% | Final Sample |
| --- | --- | --- | --- | --- | --- | --- | --- |
| Maternal Education | 23.2% | 80% | 8.2% | 3.3 | 299 | 30 | 330 |
| Age of Child | 16.6% |  | 8.5% | 1.9 | 531 | 53 | 584 |
| Wealth Status | 20.4% |  | 6.4% | 3.2 | 186 | 19 | 205 |

Double population proportion was calculated using EpiInfo ® version 7.2. The results are shown in the table above (**Table S3**).

$$N_{Fleiss} = \frac{{[Z_{\frac{\alpha}{2}}\sqrt{\left( r+1 \right)p\left( 1-p \right)}+Z_{\beta}\sqrt{rp_{0}\left( 1-p_{0} \right)+p_{1}(1-p_{1})}]}^{2}}{{r(p_{0}-p_{1})}^{2}}$$

Where;

α: The probability of type I error (significance level)

β: The probability of type II error

P_0_: The prevalence of dietary diversity in population 1

P_1_: The prevalence of dietary diversity in population 2

N_0_: Number of population 1

N_1_: Number of population 2

r: The ratio of population 2 to population 1 (N_1_:N_0_)

N_Fleiss_: Required sample size for the population 1 using Fleiss formula
N_Fleiss Total_: Required sample size for the population 1 & 2 using Fleiss formula

**Supplementary Table S2 Subgroup analysis of factors associated with the consumption of unhealthy foods and beverages among children aged 6-23 months in Mekelle, northern Ethiopia (n=567)**

|  |  | 6-11 months | | 12-17 months | | 18-23 months | |
| --- | --- | --- | --- | --- | --- | --- | --- |
|  |  | AOR | *p-value* | AOR | *p-vlaue* | AOR | *p-value* |
| Child sex | Male | Ref |  | Ref |  | Ref |  |
|  | Female | 1.28 (0.7 - 2.37) | 0.425 | 0.59 (0.27 - 1.29) | 0.183 | 1.16 (0.44 - 3.05) | 0.769 |
| Child birth order | First | Ref |  | Ref |  | Ref |  |
|  | Second | 4.09 (0.71 - 23.55) | 0.115 | 0.73 (0.09 - 5.98) | 0.773 | 2.98 (0.2 - 44.39) | 0.428 |
|  | Third & above | 4.09 (0.82 - 20.55) | 0.087 | 5.65 (0.45 - 71.03) | 0.18 | 12.31 (0.01 - 11650.63) | 0.473 |
| Maternal age | 17 - 25 | Ref |  | Ref |  | Ref |  |
|  | 26 - 35 | 0.64 (0.27 - 1.54) | 0.322 | 0.7 (0.24 - 2.07) | 0.522 | 0.96 (0.2 - 4.65) | 0.962 |
|  | > 36 | 0.82 (0.2 - 3.31) | 0.781 | 0.6 (0.09 - 3.99) | 0.593 | 0.52 (0.05 - 5.56) | 0.592 |
| Household members | 1 - 3 | Ref |  | Ref |  | Ref |  |
|  | 4* | **0.16 (0.03 - 0.86)** | **0.033** | 2.19 (0.25 - 19.15) | 0.48 | 0.23 (0.02 - 3.34) | 0.279 |
|  | 5 – 8 | 0.22 (0.05 - 1.02) | 0.053 | 0.37 (0.03 - 4.26) | 0.427 | 0.08 (0 - 74.91) | 0.473 |
| Marital status | Married | Ref |  | Ref |  | Ref |  |
|  | Other* | 0.26 (0.06 - 1.09) | 0.065 | **0.17 (0.03 - 0.94)** | **0.043** | 0.31 (0.04 - 2.4) | 0.263 |
| Maternal education years | < 8 | Ref |  | Ref |  | Ref |  |
|  | 8 – 12 | 1.92 (0.78 - 4.73) | 0.157 | 1.61 (0.51 - 5.03) | 0.416 | 0.36 (0.07 - 2) | 0.244 |
|  | > 12 | 1.49 (0.5 - 4.47) | 0.476 | 0.81 (0.19 - 3.35) | 0.766 | 0.46 (0.05 - 4.26) | 0.491 |
| Maternal occupation | Homemaker | Ref |  | Ref |  | Ref |  |
|  | Government | 1.78 (0.69 - 4.6) | 0.233 | 0.78 (0.25 - 2.41) | 0.671 | 0.6 (0.13 - 2.79) | 0.511 |
|  | Other | 1.25 (0.55 - 2.84) | 0.596 | 0.96 (0.28 - 3.25) | 0.946 | 9.4 (0.83 - 106.17) | 0.07 |
| Paternal education years | < 8 | Ref |  | Ref |  | Ref |  |
|  | 8 – 12 | 1.38 (0.51 - 3.75) | 0.525 | 1.75 (0.45 - 6.74) | 0.419 | 1.4 (0.18 - 10.93) | 0.746 |
|  | > 12 | 0.56 (0.19 - 1.61) | 0.28 | 0.88 (0.21 - 3.72) | 0.858 | 1.11 (0.13 - 9.11) | 0.925 |
| Paternal occupation | Government | Ref |  | Ref |  | Ref |  |
|  | Merchant | 1.68 (0.71 - 4) | 0.239 | 0.73 (0.25 - 2.19) | 0.58 | 2.1 (0.34 - 13.2) | 0.427 |
|  | Other | 1.02 (0.42 - 2.48) | 0.962 | 0.94 (0.3 - 2.88) | 0.907 | 1.38 (0.29 - 6.5) | 0.684 |
| Household income tercile | Low | Ref |  | Ref |  | Ref |  |
|  | Medium | 0.53 (0.25 - 1.1) | 0.087 | 0.59 (0.21 - 1.64) | 0.312 | 0.91 (0.25 - 3.24) | 0.88 |
|  | High* | **0.29 (0.13 - 0.67)** | **0.004** | 0.97 (0.32 - 2.94) | 0.962 | 0.93 (0.22 - 3.98) | 0.922 |
| Maternal internet use | Yes | Ref |  | Ref |  | Ref |  |
|  | No* | 1.25 (0.59 - 2.67) | 0.564 | **3.36 (1.06 - 10.66)** | **0.04** | 0.71 (0.14 - 3.52) | 0.673 |

*statistically significant; AOR: Adjusted Odds Ratio

**Supplementary Table S3 Sensitivity analysis using complete case analysis of factors associated with the consumption of unhealthy foods and beverages in Mekelle, northern Ethiopia**

|  |  | Imputed data (n=567) | | Complete case only (n=495) | |
| --- | --- | --- | --- | --- | --- |
|  |  | AOR | p-value | AOR | p-value |
| Child age in months | 6-11 | Ref | Ref | Ref | Ref |
|  | **12-17*** | **2.3 (1.46 - 3.64 )** | **<0.001** | **2.53 (1.55 - 4.13)** | **< 0.001** |
|  | **18-23*** | **4.14 (2.36 - 7.26 )** | **<0.001** | **4.14 (2.23 - 7.69)** | **< 0.001** |
| Child sex | Male | Ref |  | Ref | Ref |
|  | Female | 1.14 (0.76 - 1.7) | 0.531 | 1.13 (0.73 - 1.75) | 0.593 |
| Child birth order | First | Ref |  | Ref | Ref |
|  | Second | 2.5 (0.79 - 7.92) | 0.12 | **4.67 (1.02 - 21.48)** | **0.048** |
|  | **Third & above*** | **3.78 (1.19 - 11.98)** | **0.024** | **4.15 (1.09 - 15.88)** | **0.037** |
| Maternal age | 17 – 25 | Ref |  | Ref | Ref |
|  | 26 – 35 | 0.69 (0.38 - 1.24) | 0.213 | 0.92 (0.48 - 1.75) | 0.8 |
|  | > 36 | 0.67 (0.26 - 1.76) | 0.417 | 0.93 (0.33 - 2.63) | 0.892 |
| Household members | 1 – 3 | Ref |  | Ref | Ref |
|  | **4*** | **0.31 (0.1 - 0.96)** | **0.042** | **0.15 (0.03 - 0.67)** | **0.013** |
|  | **5 – 8*** | **0.28 (0.09 - 0.87)** | **0.028** | **0.22 (0.06 - 0.82)** | **0.025** |
| Marital status | Married | Ref |  | Ref | Ref |
|  | **Other*** | **0.26 (0.11 - 0.62)** | **0.002** | 0.44 (0.1 - 1.98) | 0.286 |
| Maternal education in years | < 8 | Ref |  | Ref | Ref |
|  | 8 – 12 | 1.39 (0.76 - 2.54) | 0.288 | 1.54 (0.76 - 3.11) | 0.226 |
|  | > 12 | 1.12 (0.53 - 2.35) | 0.768 | 1.19 (0.51 - 2.81) | 0.686 |
| Maternal occupation | Homemaker | Ref |  | Ref | Ref |
|  | Gov’t | 1.03 (0.55 - 1.9) | 0.935 | 1.07 (0.55 - 2.05) | 0.849 |
|  | Other | 1.39 (0.76 - 2.53) | 0.285 | 1.65 (0.83 - 3.28) | 0.154 |
| Paternal education in years | < 8 | Ref |  | Ref | Ref |
|  | 8 – 12 | 1.29 (0.64 - 2.59) | 0.471 | 1.06 (0.5 - 2.25) | 0.882 |
|  | > 12 | 0.7 (0.33 - 1.48) | 0.355 | 0.55 (0.24 - 1.28) | 0.166 |
| Paternal occupation | Government | Ref |  | Ref | Ref |
|  | Merchant | 1.34 (0.74 - 2.43) | 0.339 | 1.21 (0.65 - 2.27) | 0.546 |
|  | Other | 1.1 (0.61 - 2) | 0.753 | 1.08 (0.57 - 2.04) | 0.811 |
| Household income tercile | Low | Ref |  | Ref | Ref |
|  | Medium | 0.6 (0.36 - 1) | 0.052 | 0.68 (0.39 - 1.2) | 0.183 |
|  | **High*** | **0.49 (0.28 - 0.87)** | **0.014** | **0.55 (0.31 - 0.99)** | **0.046** |
| Maternal internet use | Yes | Ref |  | Ref | Ref |
|  | No | 1.49 (0.87 - 2.55) | 0.142 | 1.51 (0.83 - 2.74) | 0.175 |

*statistically significant; AOR: Adjusted Odds Ratio
